# Dietary tryptophan depletion in humans using a simplified two amino acid formula – a pilot study

**DOI:** 10.3402/fnr.v60.29272

**Published:** 2016-12-16

**Authors:** Maike Linden, Katrin Helmbold, Janina Kempf, Shabnam Sippas, Christian Filss, Karl-Josef Langen, Albrecht Eisert, Florian Daniel Zepf

**Affiliations:** 1 Clinic for Child and Adolescent Psychiatry, Psychosomatics and Psychotherapy, RWTH Aachen University, Aachen, Germany; 2 JARA Translational Brain Medicine, Jülich Aachen Research Alliance, Jülich, Germany; 3 Faculty of Arts and Social Sciences, Maastricht University, Maastricht, The Netherlands; 4 Section JARA-Brain, Jülich-Aachen Research Alliance (JARA), Jülich, Germany; 5 Institute of Neuroscience and Medicine (INM-3, -4, -5), Research Centre Jülich, Jülich, Germany; 6 Department of Nuclear Medicine, RWTH Aachen University Hospital, Aachen, Germany; 7 Department of Pharmacy, RWTH Aachen University, Aachen, Germany; 8 Centre and Discipline of Child and Adolescent Psychiatry, Psychosomatics and Psychotherapy, School of Psychiatry and Clinical Neurosciences & School of Paediatrics and Child Health, Faculty of Medicine, Dentistry and Health Sciences, The University of Western Australia, Perth, Australia; 9 Specialised Child and Adolescent Mental Health Services (CAMHS), Department of Health in Western Australia, Perth, Australia

**Keywords:** serotonin, amino acids, dietary tryptophan depletion, influx rate, Michaelis–Menten kinetics, humans

## Abstract

**Background:**

Acute tryptophan depletion (ATD) is a well-established dietary method in translational brain research used to briefly lower central nervous serotonin (5-hydroxytryptamine (5-HT)) synthesis. A simplified two amino acid ATD formula (ATD_PHE/LEU_) was developed while reducing the overall amount of amino acids (AAs), with the objective of administration especially in children and adolescents in future studies.

**Objective:**

This study investigated tryptophan (TRP) influx rates across the blood-brain barrier (BBB) after dietary ATD_PHE/LEU_ administration relative to the ATD Moja-De protocol that has been established for use in children and adolescents.

**Design:**

Seventy-two healthy adults (50% females) were randomized into four groups and administered ATD Moja-De, its TRP-balanced control condition (BAL), ATD_PHE/LEU_, or its respective control mixture (BAL_PHE/LEU_) in a counterbalanced, double-blind, between-subjects design. Blood samples were collected at baseline and at hourly intervals for 6 h after AA intake. Questionnaires about mood, taste, and challenge tolerance were completed at fixed time points.

**Results:**

Both challenge mixtures significantly reduced central nervous TRP influx as calculated by Michaelis–Menten kinetics relative to baseline and the respective control conditions with only mild and comparable side effects. A greater decline in TRP influx over the BBB after ATD_PHE/LEU_ administration when compared with ATD Moja-De was detected without group effects for taste, challenge tolerance, and mood. There was unintended initial short increase in plasma TRP concentrations observed after ATD_PHE/LEU_ intake, and a possible redistribution between free and protein-bound TRP triggered by protein synthesis stimulated by the ingested AAs may account for this finding. Moreover, a decline in TRP influx after BAL_PHE/LEU_ administration over a 6-h period was observed, and the large amount of PHE in the BAL_PHE/LEU_ mixture may be a possible explanation for this particular phenomenon, which could have led to an unexpected increase in displacement of TRP at the BBB in this control condition.

**Conclusions:**

This pilot study provides preliminary evidence for the possibility of lowering TRP influx as calculated by Michaelis–Menten kinetics into the brain by using a simplified ATD protocol in humans. The simplified composition of only two AAs, the lower overall AA amount, and the appropriate tolerance are characteristics of the newly developed ATD_PHE/LEU_ protocol. Future studies focusing on the effects of the ATD_PHE/LEU_ protocol and its respective control condition on CSF 5-HIAA concentrations, as well as neurochemical studies in rodents, are needed to further validate this newly developed AA mixture before definite conclusions about its usability in ATD-related research in humans, its specificity, and additional effects can be made.

Acute tryptophan depletion (ATD) is an established dietary challenge procedure used to investigate the central nervous effects of the neurotransmitter serotonin (5-hydroxytryptamine (5-HT)) in humans and rodents by a short term and reversible reduction in the brain 5-HT synthesis rate. This method has been commonly used in translational brain research for more than 25 years and is of both clinical and scientific relevance, particularly because 5-HT modulates an extensive range of physiological and behavioral functions (1). Moreover, it is known to be involved in the pathogenesis and pathophysiology of a variety of different behaviors and neuropsychiatric disorders, including affective, eating, and attention deficit hyperactivity disorders (2–13).

ATD is accomplished through the administration of a mixture of large neutral amino acids (LNAAs) lacking tryptophan (TRP). Because 5-HT is not able to pass the blood-brain barrier (BBB), it is synthesized from its physiological precursor, the amino acid (AA) TRP, in the brain. A small proportion of TRP reaches the central nervous system through passive diffusion. The majority of free TRP uses the active transport system L-1, a carrier in the capillary cell plasma membrane specialized for the transportation of all LNAAs, to cross the BBB (14). An affinity to the aforementioned AA, increased by 100–1,000 times, characterizes the BBB-specific type of the L-1 system (15). Moreover, L-systems differing in these and other properties can be found in all cell types in the human body (e.g. gastrointestinal tractus and hepatic and renal tissues). The uptake of TRP into the brain using L-1, which is saturated under physiological conditions, marks a facilitated diffusion that follows Michaelis–Menten kinetics with competitive substrate inhibition of the LNAAs at the common transporter (15–17). When orally administering a beverage of LNAAs lacking TRP, it was shown that TRP plasma concentrations decline because of increased protein synthesis in the liver, which is induced by the administered AA load. Based on preliminary findings in rats (18), this is thought to be an underlying principle of the ATD challenge procedure introduced by Young et al. because of reduced substrate availability for brain 5-HT synthesis with regard to reduced plasma TRP levels. Moreover, ATD makes use of the outlined competitive substrate inhibition, resulting in a diminished uptake of endogenous TRP into the central nervous system at L-1 (19–23). Specifically, the administered AAs compete with endogenous TRP at L-1 for uptake into the central nervous system. The synthesis of 5-HT in the brain comprises two steps: first, the hydroxylation of TRP by the tryptophan hydroxylase 2 (TPH2) into 5-hydroxytryptophan, followed by a 5-hydroxytryptophan decarboxylase catalysis in the second step, which leads to 5-HT (24, 25). TPH2 is involved in the rate-limiting step of the central nervous 5-HT synthesis pathway, with TPH2 showing a saturation of approximately 50% under physiological conditions (26). As a consequence, reduced substrate availability of TRP due to competing mechanisms at the L-1 when other AAs are administered leads to a lowered central nervous 5-HT synthesis rate. As outlined above, AA administration is thought to induce restricted 5-HT synthesis by stimulating protein synthesis in the liver with the consumption of additional TRP from plasma stores (26, 27).

The history of ATD development is characterized by a variety of modifications of the respective AA mixtures. In 1977, Concu et al. (28) applied the ATD technique to humans using 18.2 g of a TRP-free mixture (containing L-glycine, L-lysine (LYS), L-methionine (MET), L-phenylalanine (PHE), L-leucine (LEU), L-isoleucine (ILE), L-threonine (THR), and L-valine (VAL)). They detected a decline in serum TRP by 42% in healthy men (29). Moja et al. (30) also tested a similar formulation, lacking glycine, in healthy male subjects. In their study, plasma TRP decreased to ~35% of the initial level following the administration of an AA dose of 36.6 g. Most of the published ATD studies refer to a protocol introduced by Young et al. (31). Young and colleagues used a 100 g mixture of 15 amino acids (nine LNAA and six non-essential AAs) and found a 76% decrease in plasma TRP. This formulation can cause a large range of side effects (such as nausea, vomiting, headache, and dizziness), which were also reported in other ATD studies (2, 20, 32–35). Dougherty et al. (21) found that reducing the amino acid dose to only 50 g helped diminish observed side effects while implying a slightly less robust depletion when compared with the 100 g formulation.

ATD Moja-De, a modification of the formulation by Moja et al. (30), was developed in 2002 (36, 37) with the aim of establishing a body weight-adapted dosing regimen that can be used in minors. This concept does not only help to reduce side effects to a tolerable level but also allows the application of ATD Moja-De to children and adolescents as well as adults, opening further developmental research perspectives for future studies. Moreover, ATD Moja-De is the only available ATD formulation to date that has been shown to decrease brain 5-HT in mice (38).

Based on the above mentioned developments, we decided to establish a new formulation, called ATD_PHE/LEU_, for optimizing TRP depletion procedures in healthy adult humans. Once data in adult humans are obtained as proposed in this study, this formulation could potentially be used for future studies in children and adolescents. Our motivation was to introduce a new protocol for a possible later use in children and adolescents allowing a reduction of the overall AA amount and a less complex composition, leading to a solid depletion magnitude while reducing or maintaining the very low side effects established in ATD Moja-De. Hence, a new protocol was developed based on mathematical considerations and existing data that showed differing affinities of competing AAs to L-1 (16), thus impacting TRP influx over the BBB to the greatest extent. ATD_PHE/LEU_ was intended to consist of two AAs, PHE and LEU, as these show the highest affinities to the L-1 transporting enzyme of all competing LNAAs (16, 36). Therefore, based on a recent study confirming the effectiveness of a similar simplified protocol in a mouse model (12), we expected adequate stimulation of protein synthesis in the liver (thus leading to a decline in plasma TRP) and a more effective displacement of TRP by PHE and LEU at the common BBB transporter.

The primary aim of this study was to make a comparison of the new dietary depletion protocol's efficacy against that of the established ATD Moja-De, contrasting challenge conditions (ATD, ATD_PHE/LEU_) and their counterbalanced control conditions (BAL, BAL_PHE/LEU_) in healthy adults. We collected several blood samples over a defined time interval and calculated TRP influx rates across the BBB using Michaelis–Menten kinetics with a correction for multiple substrate inhibition.

## Methods

### Study design

The study employed a controlled, double-blind, between-subjects design. One of the four different AA mixtures, ATD Moja-De, BAL (a TRP-balanced AA load serving as a control condition for ATD Moja-De), ATD_PHE/LEU_ (the new ATD AA mixture), or BAL_PHE/LEU_ (the newly developed control condition for ATD_PHE/LEU_), was administered to a sample group of healthy adult subjects. Blood samples were collected at hourly intervals for 6 h. Participants also completed questionnaires on mood, challenge tolerance, and taste evaluations.

### Ethics statement

The ethics committee of the Medical Faculty of the RWTH Aachen University, Germany, evaluated and approved the study protocol. The study was carried out in accordance with the Declaration of Helsinki. All participants provided oral and written informed consent. After completion of all measurements, participants were financially compensated.

### Sample

Participants were recruited through local advertisements. A total of 72 subjects (50% females) aged between 18 and 33 years (mean age 23.60±3.08 years) participated in the study. All subjects were right-handed native German speakers who did not suffer from any physical (excluded by interview) or psychiatric disorder (evaluated by a standardized screening interview for psychiatric disorders, the SKIDPIT-light (39)). The exclusion criteria included the following: an IQ lower than 85; smoking; the use of regular medications excluding oral contraceptives; drug abuse; pregnancy; psychoorganic syndromes; schizophrenia; affective disorders; and neurological and somatic diseases such as migraines, asthma, diabetes, metabolic or hormonal disorders, allergies or obesity. The mean BMI of the participants was 22.4±2.5 kg/m^2^. Using the CFT-20R (40), the participants’ IQ (mean IQ=112±13.6) was assessed. Approximately 20 individuals were excluded by these criteria during the recruitment process. Primary pre-screening for inclusion/exclusion criteria was conducted by telephonic interview. Written informed consent was obtained after in-person screening and clarification by a doctor. Subjects verified by self-report that they had followed the instructions regarding beverage and food intake. Negative urine drug screens and pregnancy tests were required before the administration of the AA beverages. The complete descriptive data of the sample are given in Table 1.

**Table 1:** Descriptive data of the study sample

		ATD	BAL	ATD_PHE/LEU_	BAL_PHE/LEU_
Age	M±SD	23.11±2.89	23.94±3.15	23.67±2.66	23.72±3.69
	Min.	18	18	18	19
	Max.	30	32	29	33
IQ	M±SD	107.67±16.36	113.22±12.10	114.61±14.97	112.89±10.27
	Min.	80	97	76	97
	Max.	138	142	142	134
BMI	M±SD	22.90±2.53	22.18±2.81	22.21±2.52	22.25±2.41
	Min.	18.8	17.9	19.4	18.8
	Max.	27.3	29.0	29.4	26.9

Descriptive data of the study sample are divided into four conditions/groups: acute tryptophan depletion mixture Moja-De (ATD), the TRP-balanced control condition for ATD Moja-De (BAL), the new ATD amino acid mixture (ATD_PHE/LEU_), or the newly developed control condition for ATD_PHE/LEU_ (BAL_PHE/LEU_). Mean±standard deviation (M±SD), maximum (Max.), and minimum (Min.) values are listed for age, IQ, and body mass index (BMI).

### Depletion procedure

Measurements began at the same time each morning in order to avoid the influence of circadian rhythms on metabolism and central nervous 5-HT synthesis. The AA beverages were administered between 9:00 and 10:00 a.m. The study duration was approximately 6 h. The participants were instructed to abstain from food high in protein beginning at 8:00 p.m. the night before each study day, and they were not allowed to drink any beverages other than water. They received a standardized low-protein breakfast (carbohydrate 68.3 g, fat 1.13 g, protein 5.1 g, and dietary fiber 2.3 g) containing two wheat rolls as well as one small pack of jam (25 g) and honey (25 g) immediately following the administration of the AA beverages with a 20-min time frame for breakfast. The particular AA mixtures were composed by a pharmacist and delivered within an aqueous suspension. In order to reduce the risk of undesired lump formation, the crystalline amino acids were solubilized as much as possible with a low sugar substitute in an amount of 200 ml of water using a milk frother. Beverage intake period was reduced to a minimum of approximately 3 min. The sample was divided into four randomized and counterbalanced groups of 18 people with equal gender distributions (9 women and 9 men). The administration of the different AA beverages was counterbalanced for sex. Participants of each group either received ATD, BAL, ATD_PHE/LEU_, or BAL_PHE/LEU_.

The neurodietary challenge procedure ATD Moja-De was previously used successfully with children and adolescents and had few side effects due to the administration regimen that adapted dosage to body weight (26, 36–38, 41–44). The dosage per 10 kg of body weight contained L-PHE (1.32 g), L-LEU (1.32 g), L-ILE (0.84 g), L-MET (0.5 g), L-VAL (0.96 g), L-THR (0.6 g), and L-LYS (0.96 g). TRP (0.7 g per 10 kg of body weight) was added to the corresponding control condition (BAL). The newly developed AA mixture (ATD_PHE/LEU_), also dosed per 10 kg of body weight, consisted only of L-PHE (3.2 g) and L-LEU (3.0 g); BAL_PHE/LEU_, the new mixture's control condition, contained an additional dose of 0.7 g of TRP per 10 kg of body weight.

### Blood samples

Blood samples (BS) were collected using a permanent venous catheter at baseline (T_0_) and each hour (T_1_, T_2_, T_3_, T_4_, T_5_, and T_6_) after the intake of ATD, BAL, ATD_PHE/LEU_, or BAL_PHE/LEU_ (BS_0_, BS_1_, BS_2_, BS_3_, BS_4_, BS_5_, and BS_6_), resulting in seven blood samples per subject. A 9-ml Ethylenediaminetetraacetic acid (EDTA-Tube) (EDTA S-Monovette^®^, Sarstedt, Germany) and one 7.5-ml Serum-Tube (Serum S-Monovette^®^, Sarstedt, Germany) were used for sample collection each hour. To avoid falsification of the blood values, the first 4.5 ml of every sample was rejected. The collected blood samples were kept at room temperature for 20 min and centrifuged at 2,500 g for 10 min. The resulting clear supernatant was removed with a pipette and kept in 2-ml Eppendorf Tubes^®^ at −80°C until transportation to the laboratory.

For the TRP influx calculations, plasma levels of PHE, Tyrosine (TYR), and TRP were measured using enzyme-linked immunosorbent assay (ELISA) kits (Immundiagnostik AG, Bensheim, Germany) in accordance with the manufacturer's instructions. The detection of the branched-chained AAs (BCAA: LEU, ILE and VAL) concentration using a BCAA test kit (Immundiagnostik AG, Bensheim, Germany) for photometric analyses of enzyme levels in plasma and serum was carried out according to the manufacturer's instructions. Information regarding sensitivity and specificity of the employed test kits is provided in Supplementary Table 1.

### Influx calculation into the brain for TRP, TYR, and PHE

We calculated the influx of TRP across the BBB using Michaelis–Menten kinetics with a correction for multiple substrate competition, which is an established procedure in ATD-related research (16, 45). As the competitive substrate inhibition of TRP and the LNAAs at the common transporting enzyme L-1 determines the uptake of TRP into the brain, this equation provides a valid mathematical model for calculating the unidirectional TRP influx rates into the central nervous system (26, 36, 46). The equation (see Supplementary file) includes both components influencing TRP uptake: the facilitated diffusion of AAs at L-1 and passive diffusion (36). Influx rates of the competing LNAAs PHE, TYR, as well as BCAA influx were calculated using the same equation. In addition to the abovementioned influx calculations, we also provided the following ratios to ensure comparability with previous ATD studies: [TRP]/[CAA], [TYR]/[CAA], [PHE]/[CAA], and [PHE+TYR]/[BCAA+TRP] (CAA=competing amino acids). These ratios predict the entry rates of the involved amino acids into the brain and present another possible approach for evaluating ATD protocols.

### Questionnaires

#### Mood

The German version of the Positive and Negative Affect Schedule (PANAS) (47) containing 10 items for positive (PA) and 10 items for negative (NA) affect was used to measure the extent to which depletion influenced the subjects’ mood state. Participants rated the intensity of each item on a five-point Likert scale (from 1=not at all to 5=very much) at baseline and every hour before the collection of the blood samples.

#### Challenge tolerance

We developed an in-house questionnaire on the tolerance of the AA challenges to detect if the newly developed mixture caused an equal amount of side effects as the established ATD Moja-De procedure. The questionnaire contained 12 items rated from 0 (disagree) to 3 (strongly agree). For evaluation purposes, we summarized the matching items and calculated a somatic complaints score, an awakeness score, and a hunger score. The common side effects observed in previous ATD studies, namely, headache, nausea, vomiting, dizziness, and sweating (48), were included in this questionnaire (the entire questionnaire is available in the Supplementary file). Participants completed this questionnaire every hour and immediately before and after the intake of the AA beverages.

#### Taste

For taste evaluation, an in-house questionnaire consisting of six items was employed (see Supplementary file). The consistency, the sulfuric taste, the difficulty of intake, and revulsion were assessed using a scale of 0 (disagree) to 3 (strongly agree) immediately after the intake of the AA beverages.

### Statistical analysis

As primary analysis, the influence of the four different AA mixtures on TRP influx across the BBB was investigated using repeated measures analyses of variance (rmANOVAs) with treatment as the between-subjects factor, time as the within-subjects factor, and gender as a covariate in SPSS Version 21.0 for MacOS. The degrees of freedom for the ANOVA *F*-statistics were adjusted according to the Greenhouse–Geisser correction for non-sphericity (*p<*0.05). Similar analyses were performed on TYR and PHE influx across the BBB, on [TRP]/[CAA] ratio, on tolerance (the somatic complaints score, awakeness score, and hunger score), and on the PA- and NA-PANAS scores over time. In addition, separate ANOVAs, with the Greenhouse–Geisser correction, on the TRP influx for each group using time as a within-subjects factor were performed. One-way ANOVA was used to determine any significant differences in [TRP]/[CAA] ratio, in TRP, TYR, and PHE influx rates between the groups at baseline (T_0_). The influences of the different side effects (e.g. headache, nausea, vomiting, dizziness, and sweating), part of the somatic complaints score, were assessed by rmANOVAs with time as the within-subjects factor and treatment as the between-subjects factor. One-way ANOVA with gender as a covariate was employed to analyze taste differences between the four mixtures. Gaussian distribution for all dependent variables was established with the Kolmogorov–Smirnov test for each of the four groups and for the groups formed by dividing the participants by gender. The level of statistical significance was set and kept at *p*<0.05. Because of the explorative nature of this study, alpha-adjustment was not employed. *Post hoc* power analyses were performed using G*Power Version 3.1.9.2. for MacOS.

## Results

All plasma AA concentrations of TRP, PHE, TYR, and BCAA are presented in Table 2. Table 3 includes all calculated influx values for TRP, TYR, PHE, (TYR+PHE), and BCAA across the BBB.

**Table 2:** Blood concentrations of tryptophan, phenylalanine, tyrosine, and BCAA

Parameter	Mixture	Baseline (T_0_)	T_1_	T_2_	T_3_	T_4_	T_5_	T_6_
[TRP] in μmol/l	ATD	70±3	117±22	111±23	95±22	83±22	75±23	69±17
	BAL	68±3	418±22	437±23	393±22	372±22	261±23	178±17
	ATD_PHE/LEU_	66±3	107±22	110±23	111±22	108±22	105±23	97±17
	BAL_PHE/LEU_	68±3	460±22	407±23	422±22	409±22	397±23	308±17
[PHE]	ATD	51±24	376±53	484±92	393±96	419±117	313±118	242±115
	BAL	55±24	329±53	400±92	330±96	310±117	284±118	192±115
	ATD_PHE/LEU_	112±24	972±53	1236±92	1457±96	1571±117	1427±118	1182±115
	BAL_PHE/LEU_	86±24	977±53	1474±92	1816±96	1926±117	1683±118	1416±115
[TYR]	ATD	59±4	108±8	120±13	109±14	100±14	94±15	88±12
	BAL	55±4	97±8	97±13	85±14	79±14	71±15	60±12
	ATD_PHE/LEU_	59±4	132±8	170±13	193±14	204±14	197±15	189±12
	BAL_PHE/LEU_	62±4	130±8	169±13	195±14	207±14	206±15	196±12
[BCAA]	ATD	416±20	1444±53	1458±56	1374±61	1251±57	1040±64	819±47
	BAL	412±20	1278±53	1297±56	1249±61	1137±57	905±64	744±47
	ATD_PHE/LEU_	405±20	1078±53	971±56	953±61	822±57	628±64	485±47
	BAL_PHE/LEU_	400±20	1024±53	945±56	973±61	827±57	604±64	446±47

Plasma amino acid concentrations (in µmol/l) of tryptophan, phenylalanine, tyrosine, and branched-chain amino acids (BCAA; including valine, leucine, and isoleucine) after the administration of four different amino acid mixtures: ATD (acute tryptophan depletion; challenge condition), BAL (control condition for ATD), ATD_PHE/LEU_ (newly developed challenge condition), and BAL_PHE/LEU_ (control condition for ATD_PHE/LEU_). Mean values±standard error are given for the different groups (each including 18 subjects) at the seven time points of blood withdrawal (T0–T6).

**Table 3:** Calculated influx values of particular amino acids across the BBB

Parameter	Mixture	Baseline (T_0_)	T_1_	T_2_	T_3_	T_4_	T_5_	T_6_
TRP-influx	ATD	13.573±0.479	6.912±0.603	6.004±0.609	5.715±0.645	4.897±0.637	5.485±0.752	6.452±0.552
	BAL	13.313±0.479	17.190±0.603	16.533±0.609	16.821±0.645	16.864±0.637	14.122±0.752	14.236±0.552
	ATD_PHE/LEU_	12.135±0.479	3.969±0.603	3.434±0.609	3.125±0.645	2.934±0.637	3.155±0.752	3.557±0.552
	BAL_PHE/LEU_	12.483±0.479	12.449±0.603	9.483±0.609	8.828±0.645	8.435±0.637	8.939±0.752	8.191±0.552
TYR-influx	ATD	5.647±0.297	2.997±0.167	3.002±0.173	3.139±0.173	2.911±0.189	3.514±0.266	4.078±0.311
	BAL	5.338±0.297	2.054±0.167	1.866±0.173	1.877±0.173	1.921±0.189	1.959±0.266	2.523±0.311
	ATD_PHE/LEU_	5.213±0.297	1.905±0.167	2.038±0.173	1.964±0.173	2.022±0.189	2.285±0.266	2.765±0.311
	BAL_PHE/LEU_	5.464±0.297	1.515±0.167	1.530±0.173	1.501±0.173	1.582±0.189	1.859±0.266	2.348±0.311
PHE-influx	ATD	10.391±0.630	18.342±0.628	19.733±0.918	18.719±0.981	19.493±1.120	18.008±1.219	16.975±1.119
	BAL	10.819±0.630	14.706±0.628	16.091±0.918	14.764±0.981	14.540±1.120	14.522±1.219	14.088±1.119
	ATD_PHE/LEU_	12.234±0.630	26.229±0.628	28.661±0.918	30.627±0.981	31.642±1.120	30.493±1.219	28.378±1.119
	BAL_PHE/LEU_	11.978±0.630	24.250±0.628	29.411±0.918	32.387±0.981	33.428±1.120	31.321±1.219	29.325±1.119
BCAA-influx	ATD	11.968±0.453	15.582±0.448	15.112±0.501	15.502±0.570	14.340±0.544	14.018±0.612	12.901±0.435
	BAL	11.736±0.453	12.198±0.448	11.692±0.501	12.092±0.570	11.570±0.544	10.245±0.612	10.809±0.435
	ATD_PHE/LEU_	10.937±0.453	8.505±0.448	7.131±0.501	6.524±0.570	5.587±0.544	4.464±0.612	3.872±0.435
	BAL_PHE/LEU_	10.814±0.453	7.352±0.448	6.171±0.501	5.968±0.570	5.044±0.544	3.958±0.612	3.164±0.435

Calculated influx values (in nmol/min/g brain tissue) for tryptophan (TRP), tyrosine (TYR), phenylalanine (PHE), and branched-chain amino acids (BCAA; including valine, leucine, and isoleucine) across the blood-brain barrier after the administration of four different amino acid mixtures: ATD (acute tryptophan depletion; challenge condition), BAL (control condition for ATD), ATD_PHE/LEU_ (newly developed challenge condition), and BAL_PHE/LEU_ (control condition for ATD_PHE/LEU_). Mean values±standard error are presented for the different groups (each including 18 subjects) at the seven time points of blood withdrawal (T_0_–T_6_).

### TRP influx into the brain

**Fig. 1:**
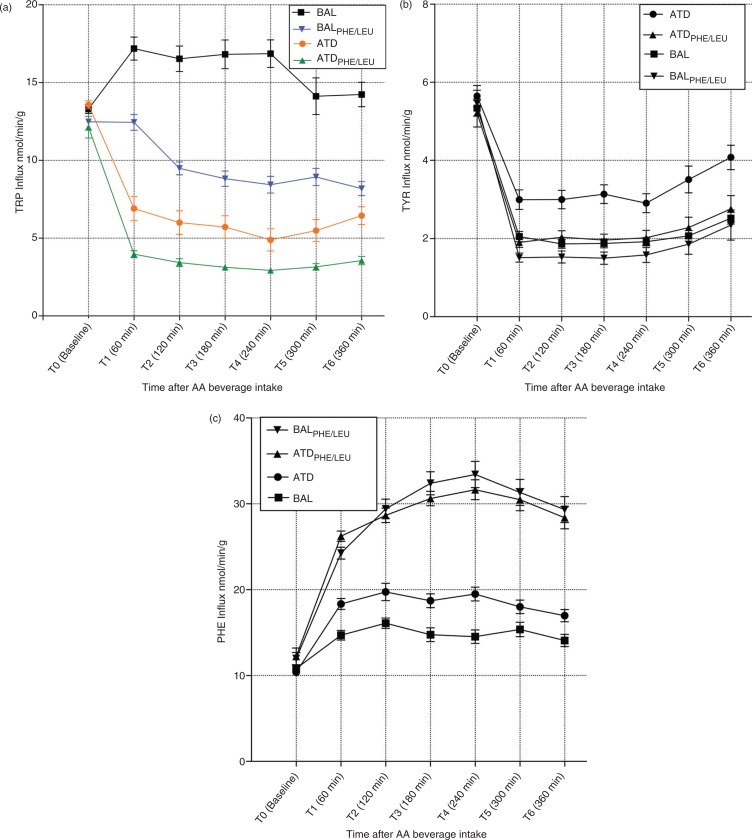
(a, b, c) Influx curves (nmol/min/g brain tissue) of tryptophan (TRP), tyrosine, and phenylalanine across the blood-brain barrier at baseline (T0) and time points T1–T6 after the intake of the ATD Moja-De amino acid (AA) mixture, its balanced control condition (BAL), the newly developed tryptophan depletion protocol ATD_PHE/LEU_, and the corresponding control condition (BAL_PHE/LEU_). Data are given as the mean values, with bars representing the standard errors.

No significant differences in TRP influx were present at baseline (T_0_) when comparing ATD, BAL, ATD_PHE/LEU_, and BAL_PHE/LEU_ (*F*_[3, 71]_=2.005; *p=n.s*.). An rmANOVA yielded main effects of time (*F*_[3.801, 258.437]_=69.972; *p<*0.001) and group (*F*_[3, 68]_=97.577; *p<*0.001) and a significant time by group interaction (*F*_[11.402, 258.437]_=25.875; *p<*0.001). Bonferroni *post hoc* analyses indicated significant differences between each of the four groups (mean TRP influx BAL>BAL_PHE/LEU_>ATD>ATD_PHE/LEU_; see Supplementary Tables 2 and 3 for detailed results). The comparison of the influx curves for all groups is given in Fig. 1a. Separate rmANOVAs with time as the within-subjects factor indicated a main effect of time for each group. Table 4 summarizes the degrees of freedom (df) and *F*- and *p*-values of these analyses. Time points of maximum TRP influx decline (T_max_), maximum decline in TRP influx in relation to baseline (Decline_max_), and overall TRP influx declines from baseline to time point T_6_ for each group are given in Table 5. Maximum decrease in TRP influx 4 h after intake was 11.9% higher for ATD_PHE/LEU_ compared with the decrease found for ATD. In contrast to ATD, ATD_PHE/LEU_, and BAL_PHE/LEU_ administration, a significant increase in TRP influx was detected in the BAL condition control group during the first 60 min after beverage administration (*p*=0.002), while no significant changes in influx rates were detected over the entire period from T_0_ to T_6_. No effect of gender was detected (*F*_[1, 67]_=2.537; *p*=n.s.). *Post hoc* power analysis showed that our study was adequately powered (power of 0.95) to detect effect sizes of 0.25 with an alpha level of 0.05 using a between-subjects repeated measures approach (ANOVA). In addition, we calculated Cohen's *d*-values as an estimate for effect sizes (*d*-value of *d*=0.24 for the comparison of TRP-influx after ATD versus BAL administration; *d*=0.14 for the comparison of ATD_PHE/LEU_ and BAL_PHE/LEU_).

**Table 4:** Results of rmANOVA on tryptophan influx values

	ATD	BAL	ATD_PHE/LEU_	BAL_PHE/LEU_
df	2.130, 36.206	3.136, 53.314	1.269, 21.579	4.057, 68.962
F	86.110	7.646	120.067	20.645
*p*	<0.001	<0.001	<0.001	<0.001

Results of separate repeated measurements analyses of variance (rmANOVAs) on tryptophan (TRP) influx across the blood-brain barrier for ATD (challenge condition), BAL (control condition for ATD), ATD_PHE/LEU_ (newly developed challenge condition), and BAL_PHE/LEU_ (control condition for ATD_PHE/LEU_), with time as the within-subjects factor. df, degrees of freedom; *F*, *F* values; *p*, statistical significance.

**Table 5:** Descriptive data of tryptophan depletion graphs

	ATD	BAL	ATD_PHE/LEU_	BAL_PHE/LEU_
T_max_ (min)	240	60	240	360
Decline_max_ (%)	63.9	29.1 (increase)	75.8	34.4
Decline_T0-T6_ (%)	52.5	6.9 (increase)	70.7	34.4

Time points of maximum tryptophan (TRP) influx decline (T_max_), maximum declines of TRP influx in relation to baseline (T_0_) (Decline_max_), and overall TRP influx declines from baseline (T_0_) to time point T_6_ (time point of last blood sample) for the groups ATD (challenge condition), BAL (control condition for ATD), ATD_PHE/LEU_ (newly developed challenge condition), and BAL_PHE/LEU_ (control condition for ATD_PHE/LEU_).

### TYR and PHE influx into the brain

An rmANOVA on TYR influx rates indicated main effects of time (*F*_[2.122, 144.270]_=226.885; *p*<0.001) and group (*F*_[3, 68]_=11.191; *p*<0.001) and a significant time by group interaction (*F*_[6.365, 144.270]_=2.255; *p*=0.038). Bonferroni *post hoc* analyses indicated that only ATD showed significant difference from the other three treatment conditions, while no significant difference could be found between the three of them. With regard to TYR influx, the curve progression for all treatment conditions was overall comparable with a strong decrease in TYR influx during the first hour (see Fig. 1b). While TYR influx after ATD administration was maximally decreased by 48% of the baseline value, influx after ATD_PHE/LEU_ displayed a decrease of 71%. Similar analyses for PHE influx values also showed main effects of time (*F*_[3.849, 261.745]_=204.575; *p*<0.001) and group (*F*_[3, 68]_=77.348; *p*<0.001) and a significant time by group interaction (*F*_[11.548, 261.745]_=2.255; *p*<0.001). Bonferroni *post hoc* analyses indicated significant differences between all groups, except of ATD_PHE/LEU_ versus BAL_PHE/LEU_ that did not present any significant results. PHE influx curves for all groups can be found in Fig. 1c. For ATD_PHE/LEU_ and BAL_PHE/LEU,_ PHE influx values showed an increase during the first 4 h, followed by only a small decrease until T_6_ (ATD_PHE/LEU_: maximum increase by 159% of baseline; BAL_PHE/LEU_: maximum increase by 179% of baseline). In comparison, a much smaller increase of PHE influx appeared after ATD (maximum increase by 89% of baseline) and BAL (increase by 49% of baseline) administration.

### [TRP]/[CAA]

**Fig. 2:**
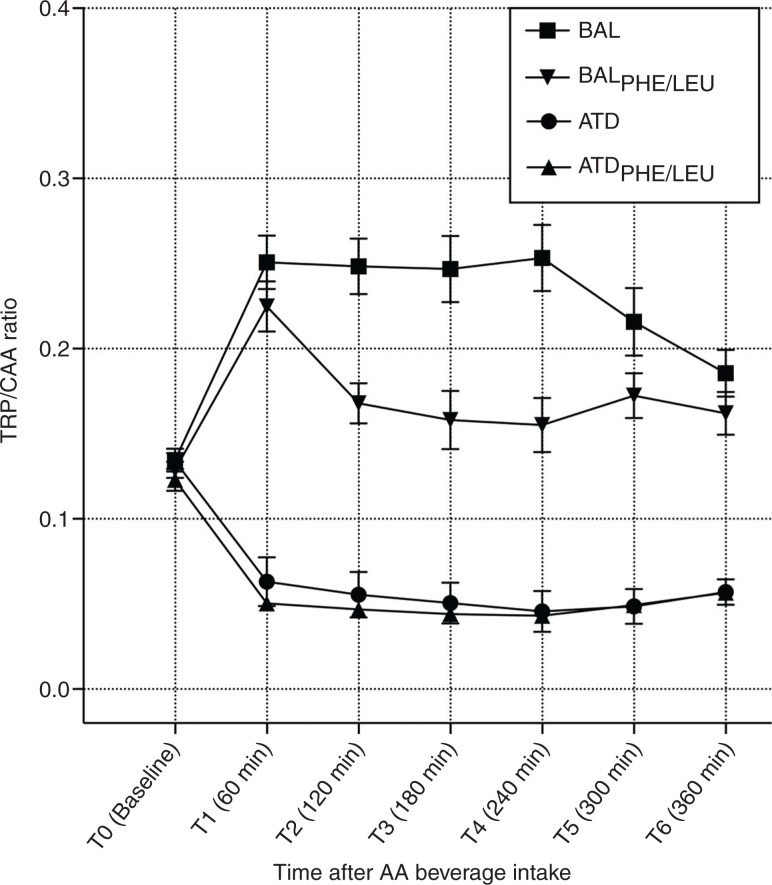
Curve progression for calculated TRP/CAA ratios from baseline (T0) to time point T6 after the intake of the ATD Moja-De amino acid (AA) mixture, its balanced control condition (BAL), the newly developed tryptophan depletion protocol ATD_PHE/LEU_, and the corresponding control condition (BAL_PHE/LEU_). Data are given as the mean values, with bars representing the standard errors.

Figure 2 shows the time course of [TRP]/[CAA] ratios for the four treatment conditions, which offers a comparable development with regard to TRP influx calculations, but with a focus on plasma concentrations. No significant differences in the ratios were present at baseline (T_0_) when comparing ATD, BAL, ATD_PHE/LEU_, and BAL_PHE/LEU_ (*F*_[3, 71]_=0.898; *p*=n.s.). An rmANOVA yielded main effects of time (*F*_[4.213, 286.489]_=5.447; *p*<0.001) and group (*F*_[3, 68]_=73.368; *p*<0.001) and a significant time by group interaction (*F*_[12.639, 286.489]_=17.960; *p*<0.001). Bonferroni *post hoc* analyses indicated significant differences between each of the four groups except of no significant result for ATD and ATD_PHE/LEU_. Maximum decrease in [TRP]/[CAA] ratio is reached 4 h after intake (T_4_) for both protocols. ATD and ATD_PHE/LEU_ reduced the ratio by 66 and 65%, respectively, when compared with baseline. Values of all calculated ratios are given in Table 6, and additional graphs for [TYR]/[CAA] and [PHE]/[CAA] ratios of all groups are presented in Supplementary Fig. S1a and S1b.

**Table 6:** Amino acid ratios

Parameter	Mixture	Baseline (T_0_)	T_1_	T_2_	T_3_	T_4_	T_5_	T_6_
[TRP]/[CAA]	ATD	0.134±0.006	0.063±0.013	0.055±0.012	0.051±0.014	0.046±0.014	0.049±0.014	0.057±0.010
	BAL	0.135±0.006	0.251±0.013	0.248±0.012	0.247±0.014	0.253±0.014	0.204±0.014	0.186±0.010
	ATD_PHE/LEU_	0.123±0.006	0.050±0.013	0.047±0.012	0.044±0.014	0.043±0.014	0.0493±0.014	0.057±0.010
	BAL_PHE/LEU_	0.129±0.006	0.225±0.013	0.168±0.012	0.158±0.014	0.155±0.014	0.172±0.014	0.162±0.010
[PHE]/[CAA]	ATD	0.094±0.043	0.232±0.037	0.320±0.078	0.293±0.077	0.329±0.087	0.308±0.117	0.254±0.139
	BAL	0.102±0.043	0.182±0.037	0.220±0.078	0.193±0.077	0.193±0.087	0.213±0.117	0.191±0.139
	ATD_PHE/LEU_	0.206±0.043	0.759±0.037	1.039±0.078	1.183±0.077	1.399±0.087	1.548±0.117	1.561±0.139
	BAL_PHE/LEU_	0.163±0.043	0.610±0.037	0.952±0.078	1.155±0.077	1.334±0.087	1.403±0.117	1.462±0.139
[TYR]/[CAA]	ATD	0.108±0.006	0.056±0.004	0.059±0.004	0.059±0.005	0.059±0.006	0.068±0.009	0.081±0.012
	BAL	0.103±0.006	0.048±0.004	0.046±0.004	0.043±0.005	0.045±0.006	0.047±0.009	0.056±0.012
	ATD_PHE/LEU_	0.105±0.006	0.063±0.004	0.075±0.004	0.079±0.005	0.086±0.006	0.103±0.009	0.124±0.012
	BAL_PHE/LEU_	0.112±0.006	0.054±0.004	0.062±0.004	0.063±0.005	0.070±0.006	0.085±0.009	0.112±0.012
[PHE+TYR]/	ATD	0.226±0.051	0.318±0.043	0.428±0.093	0.405±0.093	0.447±0.103	0.451±0.145	0.395±0.194
[BCAA+TRP]	BAL	0.229±0.051	0.250±0.043	0.289±0.093	0.255±0.093	0.257±0.103	0.285±0.145	0.270±0.194
	ATD_PHE/LEU_	0.359±0.051	0.963±0.043	1.373±0.093	1.594±0.093	1.927±0.103	2.268±0.145	2.450±0.194
	BAL_PHE/LEU_	0.313±0.051	0.756±0.043	1.188±0.093	1.452±0.093	1.723±0.103	1.882±0.145	2.096±0.194

Ratios of tryptophan, phenylalanine, tyrosine, and branched-chain amino acids (BCAA; including valine, leucine, and isoleucine) and their competing amino acids (CAA) after the administration of four different amino acid mixtures: ATD (acute tryptophan depletion; challenge condition), BAL (control condition for ATD), ATD_PHE/LEU_ (newly developed challenge condition), and BAL_PHE/LEU_ (control condition for ATD_PHE/LEU_). Mean values±standard error are given for the different groups (each including 18 subjects) at the seven time points of blood withdrawal (T0–T6).

### Taste

One-way ANOVA of the taste evaluation scores with gender as a covariate yielded no significant difference in taste perceptions between the groups (*F*_[3, 67]_=0.997; *p=*n.s.) and no effect of gender (*F*_[1, 67]_=0.102; *p*=n.s.).

### Challenge tolerance

An rmANOVA evaluation of challenge tolerance indicated a main effect of time for the analysis of the hunger score (*F*_[4.760, 314.190]_=4.457; *p=*0.001); *post hoc* testing indicated a significant increase in hunger from baseline (T_0_) to T_6_ (*p*=0.001). Similar analyses of the awakeness score also yielded a main effect of time (*F*_[4.182, 275.991]_=9.741; *p<*0.001). Within the 6 h from baseline (T_0_) to T_6_, the awakeness score decreased significantly (*p*=0.001), but participants felt most tired at T_2_. The main effects of time (*F*_[4.018, 265.208]_=5.077; *p=*0.001) and group (*F*_[3, 66]_=3.939; *p=*0.012) were detected for the somatic complaints score and indicated an increase in somatic complaints over time. Bonferroni *post hoc* analyses showed a significant difference between BAL_PHE/LEU_ and BAL administration (*p=*0.012). The average somatic complaints score related to BAL_PHE/LEU_ administration (average score 0.708±0.117) was four times greater than the values reported by the BAL condition group (average score 0.169±0.121). While the participants overall felt more unwell 2 h (T_2_) after the intake of the AA mixtures (*p*=0.022), the somatic complaints scores decreased from T_2_ to T_4_ (*p*=0.002), with only a slight, non-significant increase observed at T_6_ compared with the baseline score. At baseline, there were no significant differences in somatic complaints scores between the groups (*F*_[3, 68]_=0.169; *p=*n.s.). Analyses of each individual item in the somatic complaints score showed a comparable development of nausea. The report of nausea significantly increased from T_0_ to T_2_ (*p*=0.046) and then significantly decreased from T_2_ to T_4_ (*p*=0.014). No gender effects were detected in the hunger (*F*_[1, 65]_=0.192; *p=*n.s.), awakeness (*F*_[1, 65]_=0.769; *p=*n.s.), or somatic complaints scores (*F*_[1, 65]_=0.179; *p=*n.s.).

### Mood assessment (PANAS)

An rmANOVA of the PA score showed a main effect of time (*F*_[3.335, 220.091]_=54.110; *p<*0.001), but no significant effect of group (*F*_[3, 65]_=0.215; *p=*n.s.) or gender (*F*_[1, 65]_=0.933; *p=*n.s.). Compared with the baseline score, a decrease in the PA score of 28.5% at T_6_ was observed (*p*<0.001). The NA score also decreased (11.4% from baseline to T6; *F*_[3.198, 211.086]_=12.502; *p<*0.001, rmANOVA) regardless of gender (*F*_[1, 65]_=0.268; *p=*n.s.).

## Discussion

The results of this pilot study indicate that dietary administration of ATD_PHE/LEU_ led to a greater depletion magnitude with regard to the TRP influx across the BBB compared with the depletion observed when using the ATD Moja-De protocol, but not with regard to plasma TRP concentrations because of an unintended initial short increase in plasma TRP concentrations that was observed after ATD_PHE/LEU_ intake (see further below in this section for a discussion). The found effect on TRP influx across the BBB was observed even though ATD_PHE/LEU_, which consisted of only two AAs (PHE and LEU), was administered in a lower overall AA dose. Participants evaluated the tolerance level of the new AA challenge beverage as acceptable. No situations or events necessitating premature termination of the study occurred (such as participants vomiting during measurements or other incidents affecting ratings). Thus, several criteria for a potentially valid and effective ATD-test protocol were already achieved, while other aspects such as the observed unexpected increase in plasma TRP levels require further improvements and future studies in order to validate the findings of the current pilot study.

The altered composition of ATD_PHE/LEU_, including only two AAs, in contrast to the ATD Moja-De that includes seven different AAs, should lead to a greatly simplified production of the AA beverage. Moreover, it also enables facilitated monitoring of relevant blood parameters (i.e. AA concentrations) as their number was consequently reduced. Such monitoring was further simplified by the development and use of ELISA and BCAA test kits (Immundiagnostik AG, Bensheim, Germany). ATD_PHE/LEU_ uses the same body weight-adapted dosing regimen of the ATD AA mixtures that was first successfully applied in the ATD Moja-De protocol (36, 37). This well-tolerated dosing regimen is based on the assumption of a positive correlation between body weight and plasma TRP levels (36, 37) and, as a significant advantage, leads to a possible application for children and adolescents with fewer side effects (42–44, 49, 50).

The greater effectiveness of ATD_PHE/LEU_ in terms of a greater reduction in TRP influx across the BBB when compared with ATD Moja-De could be seen as an advantage, whereas the found effects on plasma TRP concentrations require further investigation. The reason for the detected effects on TRP influx into the brain may lie in the different affinities of the LNAAs to the common L-1 transporter. The Michaelis constant (K_M_) represents a measurement of these particular affinities. Precisely, it is indicative of the specific substrate concentration needed to achieve the half-maximal rate of turnover of an enzyme. Thus, a lower K_M_ value of a substrate is associated with its higher affinity to the related enzyme. Smith et al. (16) determined the values of transport constants for seven LNAAs, including PHE (0.011 µmol·ml^−1^) and LEU (0.029 µmol·ml^−1^), in anesthetized rats. It must be acknowledged that transferring findings from rodent research to humans is subject to limitations. However, these values for the respective transport constants can serve as a first rough estimate, and the high and significant positive correlations between TRP influx and the TRP/CAA ratio (a previous measure for the effects of ATD) are consistent with the theoretical considerations for using Michaelis–Menten kinetics to characterize TRP influx across the BBB. The high affinity of PHE and LEU to L-1, compared with all other LNAAs, may have caused the best displacement of TRP at the common transporter and therefore was possibly associated with a greater reduction of TRP influx across the BBB. By contrast, the ATD Moja-De, containing a mixture of seven different AAs with partly lower K_M_ values, was less effective with regard to the competition of the AAs against TRP, resulting in a smaller TRP depletion magnitude in terms of TRP influx across the BBB compared with the ATD_PHE/LEU_ depletion magnitude. It is possible that the use of this competitive mechanism in the respective AAs enables the potential effectiveness of TRP depletion after ATD_PHE/LEU_ administration, despite the simultaneous reduction in the overall AA dose and thus a possibly decreased stimulation of protein synthesis in the liver when compared to other ATD protocols. This could have impacted the observed findings with regard to changes in plasma TRP concentrations and a possible redistribution between free and protein-bound TRP. The observed TRP influx curves of the old and new challenge protocols over the entire study duration resulted in maximum depletion in terms of decreased TRP influx across the BBB at 4 h after intake for both challenge procedures. In particular, TRP influx correlated highly and significantly with the TRP/CAA ratio for both ATD challenge conditions used (see Supplementary Tables S5 and S6 and Fig. S2a and S2b), but the influx rates are likely to be a more adequate reflection of TRP availability in the brain as they also take the individual affinities of the different amino acids to the L-1 transport system at the BBB into account.

It is important to keep in mind that this work only claims to be a first pilot study. Therefore, the assessment of brain TRP and 5-HT concentrations after ATD_PHE/LEU_ application in rodents has to be an essential part of future research as well as PET-studies and the measurement of 5-hydroxyindoleacetic acid (5-HIAA) concentrations (the primary metabolite of 5-HT) in human cerebrospinal fluid in order to validate the effectiveness of ATD_PHE/LEU_ revealed by this study. A recent study by Sánchez et al. (12) has provided preliminary evidence for the effectiveness of simplified ATD protocols using a three-AA-formulation (PHE, LEU, and ILE); however, future studies in humans assessing cerebrospinal fluid (CSF) 5-HIAA concentrations after intake of the challenge procedure are needed.

The absence of any gender effects in the blood parameters was an expected outcome. Although central nervous 5-HT synthesis is known to significantly differ in males and females (51), we monitored peripheral TRP and LNAA plasma levels for TRP influx calculations; these parameters are not influenced by gender-specific alterations in TRP influx and related central nervous 5-HT synthesis. The performed power analyses confirmed that this study was adequately powered to detect small effects, and Cohen's d calculations indicated small estimates for effect sizes for the analyses conducted.

Specificity for depleting central nervous 5-HT synthesis (in particular with regard to possible effects on dopamine and other neurotransmitters) is always an important point to be considered with regard to all available ATD-related amino acid formulations. Previous research from our group indicated that the Moja-De protocol does not affect central nervous dopamine synthesis in mice (38). As such evidence is currently missing for the newly developed protocol ATD_PHE/LEU_, we looked at TYR and PHE plasma values and their calculated influx into the brain. The strong decrease in TYR influx after challenge application, as described above, questions the specificity of the newly developed ATD_PHE/LEU_ condition. The phenomenon of highly increasing PHE influx values in the ATD_PHE/LEU_ protocol group can be explained by its high PHE concentrations. As the strong decrease in TYR influx indicates, the high PHE dose does not mitigate the decrease in TYR influx (calculated from peripheral blood values). Whether the high PHE amount in the ATD_PHE/LEU_ mixture directly affects central nervous concentrations of TYR formation can only be answered using future neurochemical challenge studies. However, specificity for respective neurotransmitters is always a critical issue when dealing with different TRP depletion protocols. This is a general problem in ATD-related research in humans and affects all currently available ATD amino acid formulations. Badawy et al. (52) provided an approach to enhance ATD formulations’ specificity by decreasing the amount of BCAAs. In accordance with this, the reduction of the LEU amount in our newly developed ATD_PHE/LEU_ protocol might be a potential avenue for future research. An unintended rather substantial increase in plasma TRP was observed after ATD_PHE/LEU_ intake besides the fact that no TRP was ingested, and future studies are needed to investigate and replicate this unknown effect. Protein synthesis can be stimulated by the ingested amino acids, which in turn might have led to a possible redistribution between free and protein-bound TRP. Following this train of thought, the amount of free TRP possibly was slightly higher while the overall balance remained unaffected. It also needs to be outlined that TRP-related metabolites, such as quinolinic acid, kynurenic acid, and tryptamine that can be changed due to administration of the balanced mixture, can influence brain function. This stands in line with the issue of the specificity of all ATD/BAL paradigms currently available. Finally, the reason why the fasting TRP levels (T0) were higher than those mentioned in the literature is not clear but worth noting and might be specific to the investigated sample.

Another issue regarding our new ATD challenge protocol is the absence of an appropriate control condition that ‘maintains baseline values without altering the biochemical or behavioral parameters’ (52). The observed rise in TRP influx approximately 1 h after administration of the BAL control condition seems to be a logical consequence of the enhanced TRP availability because the BAL mixture contained TRP. For this particular reason, the decline in TRP influx of 34.4% after BAL_PHE/LEU_ administration over the 6-h period appears to be counterintuitive. When comparing BAL_PHE/LEU_ with its challenge condition ATD_PHE/LEU_, the administration of BAL_PHE/LEU_ led to a depletion of approximately 50% in magnitude of the depletion observed following ATD_PHE/LEU_ administration. The large amount of PHE in the BAL_PHE/LEU_ mixture may be a possible explanation for this particular phenomenon, which in turn could lead to an unexpected increase in displacement of TRP at the BBB in this control condition. To address this particular problem, either an increase in TRP or decreased levels of PHE or LEU in the control condition could be evaluated in future research in order to establish a valid control condition for ATD_PHE/LEU_.

Both protocols that were applied in this study, ATD and BAL on the one hand and ATD_PHE/LEU_ and BAL_PHE/LEU_ on the other hand, were well tolerated by the subjects. In particular, we did not observe any headaches, vomiting, dizziness, or sweating as indicated by the itemizing analyses of our somatic complaints score (see Supplementary Table S3). There were no dropouts caused by side effects. Hence, this requirement of a valid test protocol was met. Although we could not observe a significant group effect for the somatic complaints score between ATD and ATD_PHE/LEU_ indicating a reduction of side effects in the new challenge condition, we achieved the goal of maintaining the high-grade safety of the established ATD Moja-De.

The monitored increase in hunger throughout the study day most likely resulted from the 6-h abstinence from food and a breakfast overall low in nutrition. The lack of any group effect in hunger ratings supports the previous assumption. This observation justifies the necessity of the low-protein breakfast at the beginning of the study day. However, although its carbohydrate content can, to some extent, affect plasma TRP concentration and binding and levels of BCAA, it would not be justifiable to demand fasting beginning at 8 p.m. the night before the study day till 3 p.m. on the study day. Furthermore, the collection of additional behavioral, cognitive, and emotional data during this study for future analysis would have been likely to be skewed by significant fasting effects if no breakfast had been given. In this context, a variety of ATD-using studies also administer low-protein meals during the depletion procedure (53–56). In the present sample, we attribute the occurrence of increasing tiredness during the study day to external circumstances, such as the study environment and the long testing period, as opposed to a side effect of the protocol. The missing group effect can be seen as somewhat indicative for this phenomenon. Moreover, breakfast intake could also have impacted side effect ratings as assessed within the present study.

As stated above, we found an effect of group and time for the somatic complaints score. In the separated item analyses, nausea is the only side effect showing a main effect of time and displays an equal development. Although few ATD studies focused on the examination of side effects, nausea appears to be a frequent problem in these particular studies (31, 48). It is very likely that the ingestion of a substantial amount of crystalline AAs (Moja-De: 6.5 g/10 kg body weight, ATD_PHE/LEU_ 6.2 g/10 kg body weight) that remain in the stomach until dissolution in the acidic environment contributed to the reports of nausea (57). The missing group effect in somatic complaint investigations partially supports this assumption because all groups were equally affected. Moreover, *post hoc* analyses of the somatic complaints scores revealed a significant difference between BAL and BAL_PHE/LEU_ administration as a group effect with an average somatic complaints score four times greater after BAL_PHE/LEU_ intake. However, with a maximal somatic complaints score of 24 and a minimal score of 0, the very low mean value observed in this study was not indicative of the intolerability of the new control condition. A modification of the BAL_PHE/LEU_ control condition, as mentioned above, might help improve the side effect profile of BAL_PHE/LEU_.

Presently, there is no ATD-specific validated questionnaire available for somatic complaint or taste assessment. This study provides first questionnaire-based pilot data and certainly requests validation in future ATD studies. In particular, further refinement is needed regarding the gradual and sensible detection of symptom nuances.

Both positive (PA) and negative (NA) PANAS assessments decreased over the 6-h study duration. In addition to these controversial findings, no group effect was detected, which suggests that the ratings were influenced by the situation during measurements and, perhaps, not a direct consequence of altered brain 5-HT function. This is in agreement with previous findings which suggested that ATD's influence on mood was largely dependent on the susceptibility of the study sample, and this variability led to a lack of clear mood effects in many cases (58, 59). However, the assessment of mood only served as an ancillary controlling variable in this study, and these results do not significantly impact or question its major findings.

Although subjects took the amino acid mixtures without any major problems and the new mixture was well tolerated in our study, the uncomfortable palatability seen in all groups certainly requests further modification to ensure trouble-free utilization. Bitterness in general is a problematic issue in all ATD studies as it arises from the specific biochemical structure of polar and hydrophobic groups in some amino acids (60). The reduction of the overall amount and dose as well as the absence of the worst tasting AAs (i.e. MET, cysteine, and arginine) might have contributed to an easier administration of ATD_PHE/LEU_; however, improvements are still required. For this particular purpose, we suggest to add some of the conventional flavoring supplements, such as vanilla, peppermint, raspberry, and lemonade, with low sugar substitutes to our challenge procedure.

The between-subjects design is a limitation of this study. Behavioral paradigms conducted as a part of this study (the findings will be the subject of further publications) did not allow for a within-subject repeated measures design due to possible learning effects; a within-subjects design could be used in further investigations.

Regarding the use of ATD_PHE/LEU_ in behavioral studies, the high amount of LEU might present a problem, in particular as LEU is known to alter glutamate formation within the leucine-glutamate cycle (61, 62). Since LEU might also affect various other parameters, a separate analysis of LEU blood values would be essential to achieve better knowledge of ATD_PHE/LEU_'s metabolic consequences. In this study, we chose the assessment of the BCAAs within a sum parameter for initial investigations as it provides a cost-saving alternative. Moreover, a further aspect that should be considered in future studies would be to add caffeine withdrawal as an additional exclusion criterion since caffeine has been shown to induce alterations in serotonergic neurotransmission (63, 64).

The ATD method as a serotonergic challenge tool was criticized by Van Donkelaar et al. in a review article (65). Specifically, the authors advised caution in interpreting the selective serotonergic effect of ATD-related results, postulating a contribution by various other non-serotonergic mechanisms to diminished 5-HT metabolism (e.g. alterations in cerebrovascular blood flow, stress influence, and metabolic effects). Furthermore, the missing possibility of direct attribution of ATD-induced behavioral alterations to changes in 5-HT neuronal activity was noted. However, many of the suggested mechanisms and the related hypotheses were based on limited data and primarily indirect conclusions. Overall, there is sufficient and convincing evidence that ATD leads to diminished central nervous 5-HT synthesis. In a rodent model, Biskup et al. (38) demonstrated substantially impaired brain 5-HT synthesis after ATD Moja-De administration. Furthermore, they found a lowered content of 5-HIAA, which is the primary metabolite of 5-HT, and a consequently reduced 5-HT release. As summarized and outlined by Crockett et al. (66), all of the alternative mechanisms suggested in the aforementioned review would lead to broad attentional and executive impairments that have not been found in any ATD study involving humans. In general, we support the need for further research to gain a solidly grounded understanding of the underlying mechanisms of ATD because the extent of contributions of additional mechanisms cannot be reliably estimated.

In conclusion, the greater decline in TRP influx rates across the BBB in healthy adults after the dietary administration of the newly developed simplified ATD protocol, ATD_PHE/LEU_, relative to the established ATD Moja-De mixture, was the major finding of this study. The altered composition of ATD_PHE/LEU_, composed of only two LNAAs (PHE and LEU), enabled the reduction in the overall AA amount, had acceptable tolerance, and ensured simplified blood value monitoring due to the reduced components. Nevertheless, this work only represents a first pilot study. Future research is warranted not only to replicate the present findings (optimally a within-subject repeated measures design) but also to improve beverage palatability, its specificity for central nervous 5-HT depletion, and to develop a more suitable balanced control condition (BAL_PHE/LEU_) without significant TRP-related depletion effects. The direct proof of reduced brain 5-HT synthesis in a rodent model (i.e. 5-HT concentrations in brain tissue) would be beneficial, as well as investigations of 5-HIAA levels in human cerebrospinal fluid in order to further validate the newly developed ATD_PHE/LEU_ mixture. Moreover, as an additional step, ATD_PHE/LEU_ requires further study before administering this mixture to children and adolescents can be considered. If the remaining questions with regard to changes in plasma TRP levels, aspects of specificity, and the respective control condition are clarified, the simplified composition of ATD_PHE/LEU_ might have the potential to become an advantageous alternative to the currently available ATD Moja-De protocol for the investigation of the physiological and behavioral effects of reduced central nervous 5-HT synthesis in translational research. However, at this stage, the newly developed ATD_PHE-LEU_ mixture needs further validation and is currently not suitable to specifically study central nervous 5-HT function in humans, and at this point in time it cannot replace the ATD Moja-De ATD protocol.

## Supplementary Material

Dietary tryptophan depletion in humans using a simplified two amino acid formula – a pilot study
