# Supplementary material for: Dietary tryptophan depletion in humans using a simplified two amino acid formula – a pilot study
Source: Food Nutr Res. 2016 Dec 16;60:10.3402/fnr.v60.29272. doi: 10.3402/fnr.v60.29272 (PMC5165058; doi:10.3402/fnr.v60.29272)
Supplement: Dietary tryptophan depletion in humans using a simplified two amino acid formula – a pilot study [file FNR-60-29272-s001.pdf]

SUPPLEMENTARY ONLINE MATERIALS

**Table S1: Sensitivity and specificity of the employed ELISA test kits**		
	**Sensitivity** (Detection limit [µmol/l])	**Specificity** (*Cross reactivity)*
**Tryptophan**	7	5-HTP < 0,5% (5-Hydroxy-Tryptophan)<br>L-Phenylalanine < 0,1%<br>L-Tyrosine < 0,1%
**Phenylalanine**	3,75	L-Tyrosine < 0,02%
**Tyrosine**	3,6	L-Phenylalanine < 2%<br>L-Tryptophan < 0,5%
**BCAA**	5,8	No cross reactivity with L-Methionine was found.

<u>Formula for TRP influx calculations across the blood-brain barrier:</u>

$$\text{TRP influx} = \frac{V_{max}C}{(K_m[1 + \sum(C_i/K_i)] + C)} + K_dC$$

C = TRP plasma concentration

$\mathbf{V_{max}}$ = Maximum rate of conversion

$\mathbf{K_m}$ = Michaelis constant

$\mathbf{C_i}$ = Plasma concentration of competing amino acids (TRP, PHE, TYR, BCAA)

$\mathbf{K_i}$ = Affinity constants of competing amino acids (TRP, PHE, TYR, BCAA)

$\mathbf{K_d}$ = Diffusion constant

**Table S2: Mean TRP Influx values of the four sample groups**	
**Group**	**Mean TRP Influx** (nmol/min/g)
ATD	7.005
BAL	15.583
$ATD_{PHE/LEU}$	4.616
$BAL_{PHE/LEU}$	9.830

**Table S3: Bonferroni post-hoc analyses rmANOVA between the four sample groups**		
**df**		3, 68
**F**		97.577
**Group**	**Group**	***p*-value**
ATD	BAL	< 0.001
	$ATD_{PHE/LEU}$	0.004
	$BAL_{PHE/LEU}$	0.001
BAL	ATD	< 0.001
	$ATD_{PHE/LEU}$	< 0.001
	$BAL_{PHE/LEU}$	< 0.001
$ATD_{PHE/LEU}$	ATD	0.004
	BAL	< 0.001
	$BAL_{PHE/LEU}$	< 0.001
$BAL_{PHE/LEU}$	ATD	0.001
	BAL	< 0.001
	$BAL_{PHE/LEU}$	< 0.001

**Table S4: Results of the item analyses of the somatic complaints score**								
**Item**	**Mixture**	**Baseline (T_0)**	**T_1**	**T_2**	**T_3**	**T_4**	**T_5**	**T_6**
**Health**	**ATD**	2.94 ± 0.04	2.94 ± 0.11	2.83 ± 0.11	3.00 ± 0.06	3.00 ± 0.09	3.00 ± 0.07	3.00 ± 0.09
	**BAL**	3.00 ± 0.04	2.83 ± 0.11	2.94 ± 0.11	3.00 ± 0.06	2.83 ± 0.09	3.00 ± 0.07	3.00 ± 0.09
	**$ATD_{PHE/LEU}$**	2.94 ± 0.04	2.94 ± 0.11	2.89 ± 0.11	2.94 ± 0.06	2.94 ± 0.09	2.89 ± 0.07	2.83 ± 0.09
	**$BAL_{PHE/LEU}$**	3.00 ± 0.04	2.72 ± 0.11	2.67 ± 0.11	2.72 ± 0.06	2.94 ± 0.09	2.72 ± 0.07	2.72 ± 0.09
**Headache**	**ATD**	0.11 ± 0.09	0.00 ± 0.05	0.22 ± 0.10	0.11 ± 0.07	0.00 ± 0.06	0.00 ± 0.06	0.11 ± 0.10
	**BAL**	0.06 ± 0.09	0.06 ± 0.05	0.00 ± 0.10	0.06 ± 0.07	0.06 ± 0.06	0.06 ± 0.06	0.06 ± 0.10
	**$ATD_{PHE/LEU}$**	0.06 ± 0.09	0.06 ± 0.05	0.17 ± 0.10	0.06 ± 0.07	0.11 ± 0.06	0.06 ± 0.06	0.17 ± 0.10
	**$BAL_{PHE/LEU}$**	0.11 ± 0.09	0.06 ± 0.05	0.17 ± 0.10	0.11 ± 0.07	0.11 ± 0.06	0.11 ± 0.06	0.28 ± 0.10
**Dizziness**	**ATD**	0.00 ± 0.08	0.06 ± 0.08	0.06 ± 0.09	0.00 ± 0.03	0.00 ± 0.04	0.00 ± 0.05	0.00 ± 0.04
	**BAL**	0.17 ± 0.08	0.06 ± 0.08	0.11 ± 0.09	0.00 ± 0.03	0.00 ± 0.04	0.00 ± 0.05	0.00 ± 0.04
	**$ATD_{PHE/LEU}$**	0.00 ± 0.08	0.00 ± 0.08	0.00 ± 0.09	0.00 ± 0.03	0.00 ± 0.04	0.06 ± 0.05	0.00 ± 0.04
	**$BAL_{PHE/LEU}$**	0.00 ± 0.08	0.22 ± 0.08	0.28 ± 0.09	0.06 ± 0.03	0.11 ± 0.04	0.11 ± 0.05	0.11 ± 0.04
**Sweating**	**ATD**	0.00 ± 0.03	0.00 ± 0.03	0.00 ± 0.03	0.00 ± 0.04	0.00 ± 0.03	0.00 ± 0.03	0.00 ± 0.00
	**BAL**	0.00 ± 0.03	0.00 ± 0.03	0.00 ± 0.03	0.00 ± 0.04	0.00 ± 0.03	0.00 ± 0.03	0.00 ± 0.00
	**$ATD_{PHE/LEU}$**	0.00 ± 0.03	0.00 ± 0.03	0.06 ± 0.03	0.00 ± 0.04	0.00 ± 0.03	0.06 ± 0.03	0.00 ± 0.00
	**$BAL_{PHE/LEU}$**	0.06 ± 0.03	0.06 ± 0.03	0.00 ± 0.03	0.11 ± 0.04	0.06 ± 0.03	0.00 ± 0.03	0.00 ± 0.00
**Nausea**	**ATD**	0.00 ± 0.03	0.44 ± 0.15	0.17 ± 0.12	0.00 ± 0.04	0.00 ± 0.00	0.00 ± 0.10	0.00 ± 0.08
	**BAL**	0.00 ± 0.03	0.11 ± 0.15	0.17 ± 0.12	0.06 ± 0.04	0.00 ± 0.00	0.00 ± 0.10	0.00 ± 0.08
	**$ATD_{PHE/LEU}$**	0.00 ± 0.03	0.28 ± 0.15	0.22 ± 0.12	0.00 ± 0.04	0.00 ± 0.00	0.28 ± 0.10	0.17 ± 0.08
	**$BAL_{PHE/LEU}$**	0.06 ± 0.03	0.56 ± 0.15	0.33 ± 0.12	0.06 ± 0.04	0.00 ± 0.00	0.17 ± 0.10	0.11 ± 0.08

Legend to Tab. S4: Results of the itemizing analyses of the somatic complaints score after the administration of four different amino acid mixtures: ATD (Acute Tryptophan Depletion; challenge condition), BAL (control condition for ATD), $ATD_{PHE/LEU}$ (newly developed challenge condition) and $BAL_{PHE/LEU}$ (control condition for $ATD_{PHE/LEU}$). Mean scores ± standard error are presented for the different groups at the seven time points of completion of the tolerance questionnaire (T_0-T_6). Self-evaluation of headache, dizziness, sweating, nausea and feeling of health represent the included items.

**Table S5: Results of correlation analysis between TRP influx and TRP/CAA ratio for ATD Moja-De**							
	**Baseline T_0**	**T_1**	**T_2**	**T_3**	**T_4**	**T_5**	**T_6**
**r**	0.859	0.936	0.911	0.904	0.924	0.912	0.843
***p*-value**	< 0.001	< 0.001	< 0.001	< 0.001	< 0.001	< 0.001	< 0.001

Legend to Tab. S5: Results of Pearson correlation analysis between tryptophan influx and TRP/CAA ratio for ATD Moja-De over time. Correlation coefficient (r) and statistical significance (*p*-value) are included for the seven time points of measurement (T_0-T_6).

**Table S6: Results of correlation analysis between TRP influx and TRP/CAA ratio for $ATD_{PHE/LEU}$**							
	**Baseline T_0**	**T_1**	**T_2**	**T_3**	**T_4**	**T_5**	**T_6**
**r**	0.827	0.928	0.962	0.970	0.976	0.979	0.980
***p*-value**	< 0.001	< 0.001	< 0.001	< 0.001	< 0.001	< 0.001	< 0.001

Legend to Tab. S6: Results of Pearson correlation analysis between tryptophan influx and TRP/CAA ratio for $ATD_{PHE/LEU}$ (newly developed challenge condition) over time. Correlation coefficient (r) and statistical significance (*p*-value) are included for the seven time points of measurement (T_0-T_6).

Figure S1 ***a,b***: Curve progression for calculated TYR/CAA and PHE/CAA ratios from baseline (T0) to time point T6 after the intake of the ATD Moja-De amino acid (AA) mixture, its balanced control condition (BAL), the newly developed tryptophan depletion protocol $ATD_{PHE/LEU}$ and the corresponding control condition ($BAL_{PHE/LEU}$). Data are given as the mean values with bars representing the standard errors.

a)

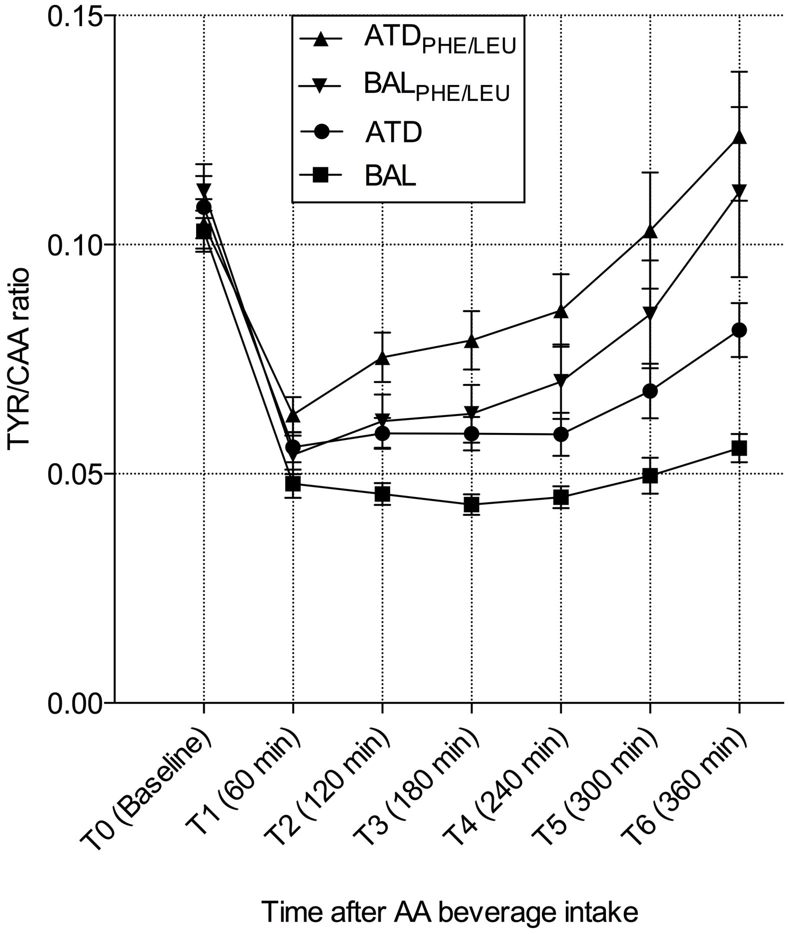

b)

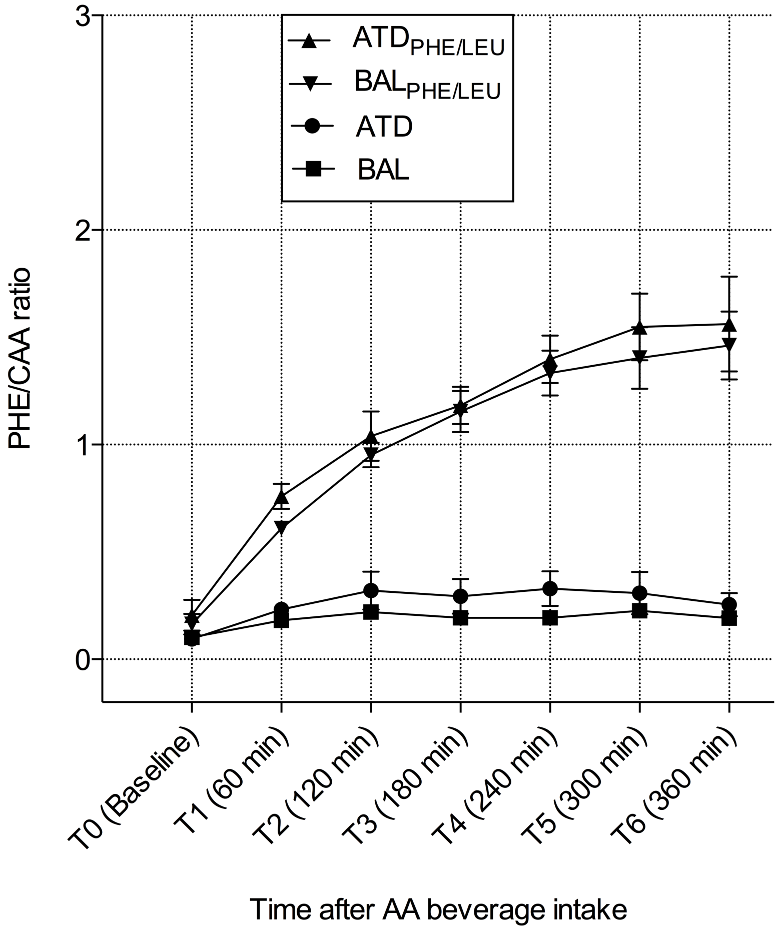

ATD PHE/LEU
BAL PHE/LEU
ATD
BAL
PHE/CAA ratio
3
2
1
0
T0 (Baseline)
T1 (60 min)
T2 (120 min)
T3 (180 min)
T4 (240 min)
T5 (300 min)
T6 (360 min)
Time after AA beverage intake

Figure S2 *a,b*: Curve progression for the percentage in reduction (%) calculated for the TRP influx and TRP/CAA ratio from baseline (T0) to time point T6 after the intake of the ATD Moja-De amino acid (AA) mixture and its balanced control condition (BAL, Figure S2 a), and for the newly developed tryptophan depletion protocol $ATD_{PHE/LEU}$ and the corresponding control condition ($BAL_{PHE/LEU}$, Figure S2 b). Data are given as the mean values with bars representing the standard errors.

a)

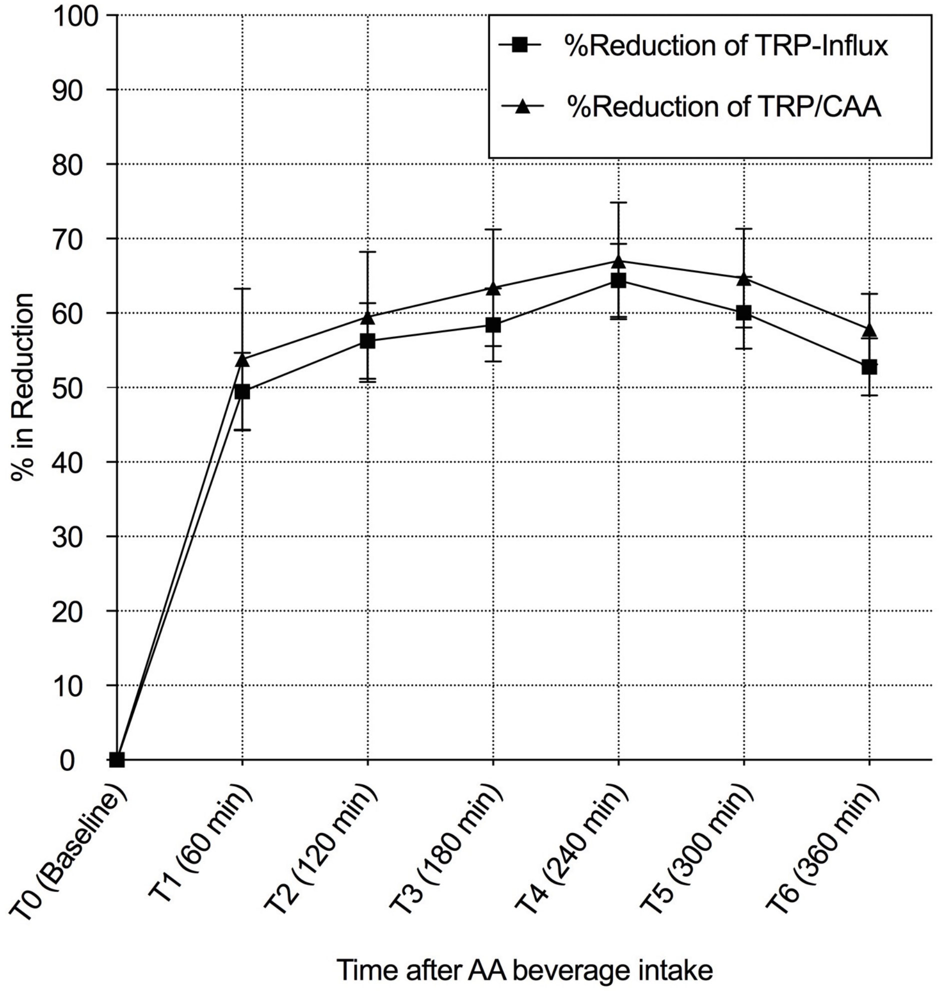

b)

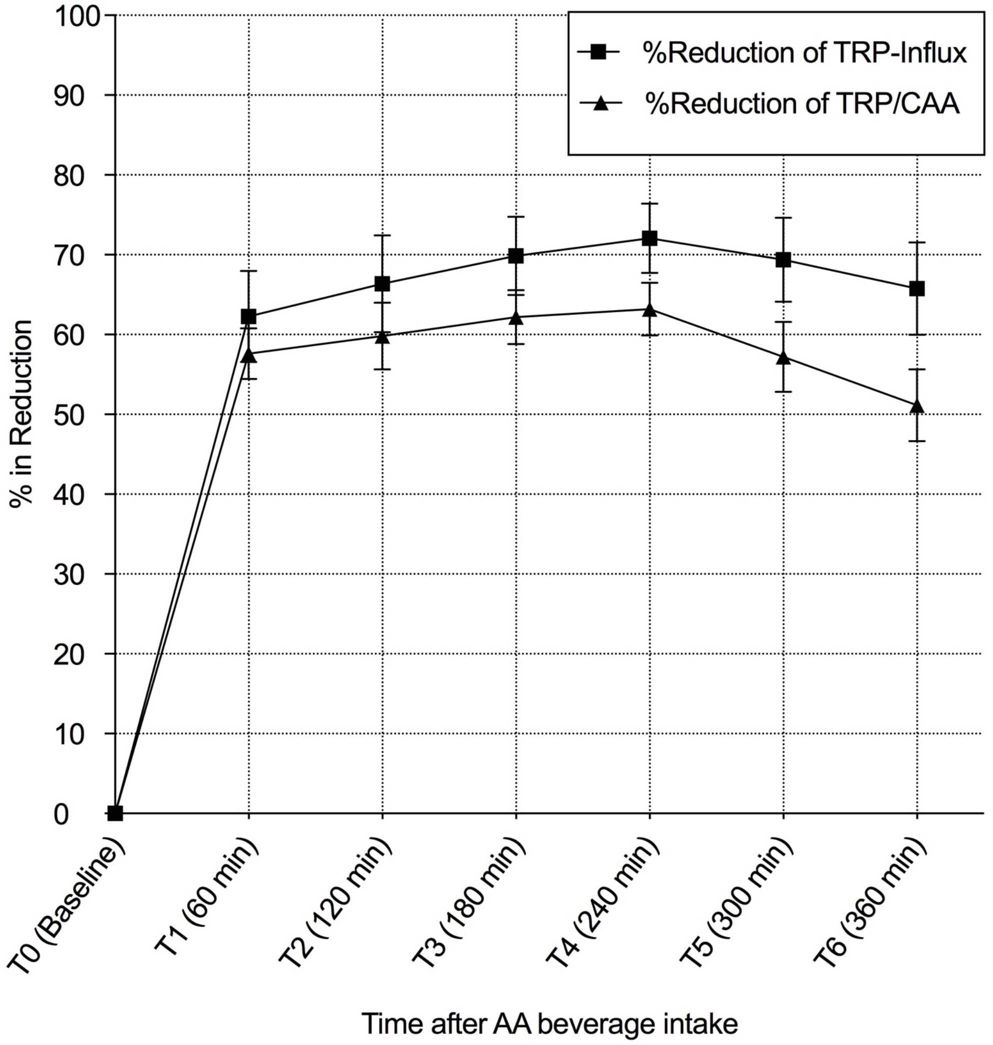

100
90
80
70
60
50
40
30
20
10
0
% in Reduction
%Reduction of TRP-Influx
%Reduction of TRP/CAA
T0 (Baseline)
T1 (60 min)
T2 (120 min)
T3 (180 min)
T4 (240 min)
T5 (300 min)
T6 (360 min)
Time after AA beverage intake

Subject Code: **Time:**

Questionnaire on Beverage Taste and Tolerance

Below, you find several statements that refer to the taste and tolerance of the amino acid beverage administered before. Please assess each statement as precise as possible by ticking the appropriate box most appropriate for you at the moment of evaluation:

0 disagree
1 mildly agree
2 agree
3 strongly agree

There are no correct or wrong answers. We are only interested in your personal opinion.

1.) Taste

	disagree	mildly agree	agree	strongly agree
1) It was possible to drink the amino acid mixture like a regular beverage (e.g., a milk shake).	0	1	2	3
2) The intake of the amino acid mixture was unpleasant for me.	0	1	2	3
3) The consistency of the beverage made it difficult to swallow.	0	1	2	3
4) The amino acid mixture tastes like sulfur.	0	1	2	3
5) The intake of the beverage triggered a feeling of disgust.	0	1	2	3
6) Usually, I do not have any problems regarding the oral administration of medicine (e.g., cough syrup, fizzy tablets).	0	1	2	3

2.) Tolerance

	disagree	mildly agree	agree	strongly agree
1) I feel healthy.	0	1	2	3
2) I have a headache at the moment.	0	1	2	3
3) The intensity of the headache would interfere with my daily activities.	0	1	2	3
4) I am suffering from severe nausea at the moment.	0	1	2	3
5) The intensity of the nausea would interfere with my daily activities.	0	1	2	3
6) The intensity of the nausea makes me feel the urge to vomit.	0	1	2	3
7) I feel dizzy at the moment.	0	1	2	3
8) I feel awake at the moment.	0	1	2	3
9) Usually I do not feel tired in this part of the day.	0	1	2	3
10) I am sweating much more than usual.	0	1	2	3

11) I am hungry.	0	1	2	3
12) My hunger is abnormally great for this part of the day.	0	1	2	3
